# Barrier genes are associated with preterm birth

**DOI:** 10.3389/fmed.2025.1580877

**Published:** 2025-06-23

**Authors:** Kuan-Ru Chen, Shih-Kai Chu, Pao-Lin Kuo

**Affiliations:** ^1^Department of Medical Research, E-DA Hospital, I-Shou University, Kaohsiung, Taiwan; ^2^Department of Statistics, National Taipei University, New Taipei City, Taiwan; ^3^Department of Obstetrics and Gynecology, National Cheng Kung University Hospital, National Cheng Kung University, Tainan, Taiwan; ^4^Department of Obstetrics and Gynecology, Kaohsiung Chang Gung Memorial Hospital, Chang Gung University College of Medicine, Kaohsiung, Taiwan; ^5^Department of Obstetrics and Gynecology, Jen-Ai Hospital, Taichung, Taiwan

**Keywords:** preterm birth, NOTCH1, maternal barrier genes, genome-wide association studies, genetic variant

## Abstract

**Background:**

Biological barriers are essential for maintaining integrity and function and preventing microbial invasion. Maternal barrier dysfunction may play a role in preterm birth (PTB). However, the link between maternal barrier function and PTB is still unknown. This study aims to identify genetic evidence supporting the role of maternal barrier genes in PTB risk.

**Methods:**

We examined 201 barrier-related genes to assess their association with PTB susceptibility. We utilized the FinnGen study, published literature's whole-genome sequencing (WGS) summary statistics and Early Growth Genetics (EGG) meta-analysis to identify the maternal barrier gene associated with PTB.

**Results:**

Findings from the analysis of the maternal genome highlighted several barrier genes (*NOTCH1, LAMA4, F11R, MAGI1, MAGI2, TJP1, PARD3, CLDN10, CLDN14, CLDN15, GRHL3, CGNL1, LAMB2, RHOA*, and *LRP5*) associated with PTB. Notably, *NOTCH1* was supported by at least two independent genomic datasets.

**Conclusion:**

The established roles of *NOTCH1* in vascular barrier function, angiogenesis, decidualization, intestinal epithelial barrier, and inflammation support its mechanistic involvement. Our research enhances our understanding of maternal barrier genes linked to PTB, providing valuable insights for future prevention and intervention strategies.

## 1 Introduction

Preterm birth (PTB) refers to the birth of a baby before completing 37 weeks of gestation (1). Epidemiological evidence indicates that PTB occurred in ~9.9% of all live births worldwide in 2020 (1). Unfortunately, ~70% of PTBs are spontaneous PTBs (sPTBs; including preterm prelabor rupture of membranes and idiopathic PTB), and remain poorly understood, with limited tools available for early identification or prevention (2). The degree of prematurity is directly proportional to the risks of mortality and morbidity (1). Moreover, PTB is associated with increased risks of long-term health and neurodevelopmental problems (3). The etiology of PTB is intricate and remains to be further explored. Cumulative evidence indicates that maternal medical disorders, antenatal risk factors, inflammatory diseases, genetic predispositions, socioeconomic factors, and environmental factors are associated with the risk of PTB (2, 4).

Biological barriers are crucial for maintaining their integrity and function, as well as preventing microbial invasion (5). Various organs possess different biological barriers, such as the skin, the intestine, the reproductive system, the lung, the central nervous system, the placental villi, and the cervix (5, 6). Several inflammatory disorders are linked to barrier dysfunction, including inflammatory bowel diseases (IBD) (7), allergic diseases (8), atopic dermatitis (9), central nervous system disorders (10), and infections. Inflammatory processes are hypothesized to play an important role in PTB, and the origin of inflammation may be due to infection or sterile inflammation (7). Approximately 60% of PTBs could be ascribed to sterile inflammation (7). Emerging evidence has linked maternal IBD (8), allergic diseases (asthma, allergic rhinitis, allergic conjunctivitis, food allergy, drug allergy, and contact dermatitis) (9), systemic maternal infections, and bacterial vaginosis (10) with risks for PTB. Barrier-related genes are those that regulate the structural and functional integrity of biological barriers. Most of these genes encode junctional or structural proteins (e.g., *CLDN* and *TJP1*), while others are involved in signaling pathways (e.g., the Wnt signaling pathway) or tissue-specific functions (e.g., mucins in the intestine and galectins in the reproductive tract). However, whether barrier dysfunction plays a role in preterm is still unknown. Thus, investigating the correlation between maternal barrier function and PTB is particularly interesting in light of these findings.

It is crucial to note that women with a history of PTB in the past are at high risk for recurrent PTB (11). These findings provide evidence of genetic predisposition to PTB. Based on epidemiological research, sPTB is influenced by both maternal and fetal genomes, but predominantly by the maternal genome (12–14). Genome-wide association studies (GWAS) (15–19), whole-exome sequencing (20), and whole-genome sequencing (WGS) (21) studies have indicated that genetic variants in maternal genomes contribute to the risk of PTB. Previous studies have identified over 750 single-nucleotide polymorphisms (SNPs) in more than 240 genes in the maternal and fetal genomes that may be associated with PTB or gestational duration at birth (2, 22). These genes involved in tissue remodeling, vascular, endothelial, metabolic, inflammatory, and immune processes are implicated (2, 22). Together, these genetic approaches can be used to confirm known associations of genetic variants and/or discover novel genetic variants.

In the present study, we hypothesized that impaired maternal barrier function may contribute to PTB. To this end, we utilized available data sources, including the FinnGen study, WGS summary statistics from published literature, and the EGG meta-analysis, to identify evidence for the involvement of maternal barrier genes in susceptibility to PTB.

## 2 Materials and methods

### 2.1 The study design

**Figure 1:**
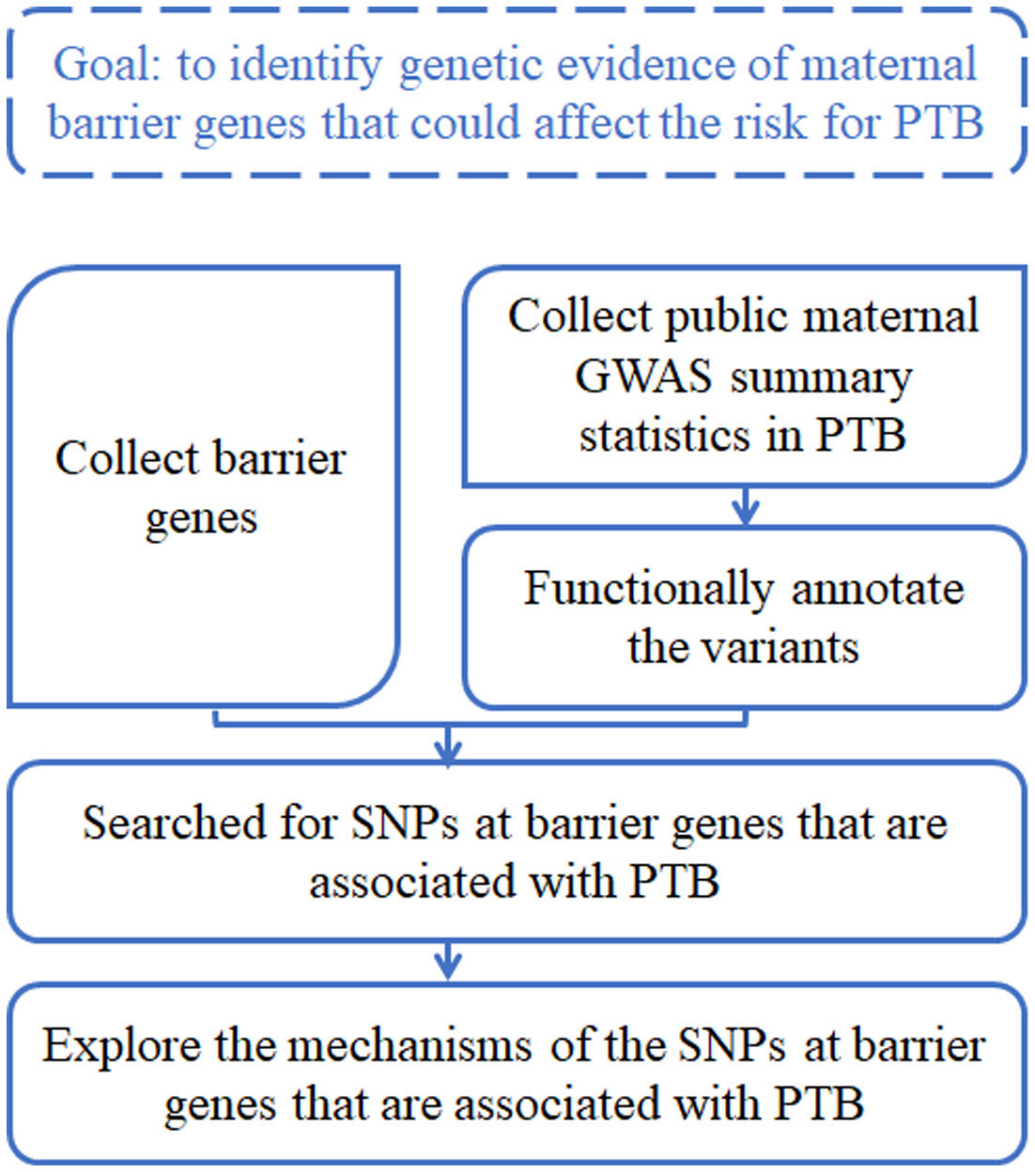
The design and workflow of this study.

The goal of this study was to identify genetic evidence of maternal barrier genes that could affect the risk for PTB. The analysis workflow was briefly described as follows (Figure 1), and the details were shown in respective sections. First, we collected a list of barrier genes from the literature search. Second, we surveyed and collected public maternal GWAS summary statistics in PTB, and functionally annotated the variants using computational tools. Third, we searched for SNPs at barrier genes that were associated with PTB. Fourth, we explored the mechanisms of those SNPs at barrier genes that are associated with PTB.

### 2.2 Barrier genes

We collected 201 barrier-related genes from the literature (Supplementary Table S1). Of these, 22 genes associated with epidermal malignancy were categorized into structural components, microenvironmental factors, and differentiation-related groups (23). A total of 146 genes related to intestinal epithelial barrier dysfunction were identified, including those involved in the mucus layer, tight junctions, adherens junctions, desmosomes, hemidesmosomes, cytoskeleton, extracellular matrix, and regulatory proteins (24). A total of 12 genes associated with the blood–brain barrier were classified as central nervous system endothelial cell genes involved in angiogenesis and barriergenesis (25). In total, 19 genes encoding galectins were included based on their role in the vaginal microenvironment as a defense barrier (26), along with two additional barrier-related genes (27, 28).

### 2.3 Datasets

We surveyed three existing maternal GWAS studies in PTB. The first study was the PTB GWAS results in FinnGen (29). FinnGen is a research project that combines genotype data from Finnish biobanks and digital health record data from Finnish health registries (https://www.finngen.fi/en) to provide new insights into disease genetics, and it has conducted a GWAS of 1,932 diseases in its 224,737 participants. We downloaded the PTB GWAS results from FinnGen release 8, which included 20,153,666 variants and a sample size of 7,678 cases and 148,153 controls. It was a maternal GWAS study, where the cases were females with a history of PTB, and the controls were females without a history of PTB. The second study aimed to identify molecular characteristics of PTB using multi-omic data (21). It used a cohort of 791 family trios from various ancestries, of which 270 had PTB. The study integrated WGS data from the fathers, mothers, and newborns in these family trios and RNA-seq gene expression and DNA methylation data from maternal blood samples and gathered comprehensive clinical information concerning pregnancy, delivery, and newborn health. We downloaded their released maternal GWAS results, which included the variants showing FDR < 0.1. The third study was a meta-analysis of results from multiple PTB GWASs (19), with a total sample size of 15,419 cases and 217,871 controls. The summary statistics were derived from maternal GWASs, where the cases consisted of females with a history of PTB, and the controls were females without a history of PTB. The data were downloaded from the website of the Early Growth Genetics (EGG) Consortium (http://egg-consortium.org).

### 2.4 Variant functional annotation of GWAS summary statistics

We searched for SNPs at barrier genes that are associated with PTB. For studies that released full GWAS summary statistics (i.e., the FinnGen and EGG datasets), the variant annotation was applied by the Functional Mapping and Annotation (FUMA) of the GWASs web application (30), where the SNPs showing *p*-value < 1 × 10^−5^ could be lead SNPs for further analysis. The obtained data of the published literature's WGS summary statistics were not full GWAS summary statistics and included only the variants showing FDR < 0.1, so we used the threshold of FDR-adjusted *p*-value < 0.1 for this dataset. We used a less stringent threshold of *p*-values, often termed “suggestive threshold,” to increase discovery power (31, 32) for understanding what possible mechanisms behind the associations between the variants and PTB. Existing studies have used the suggestive threshold in the GWAS and found valuable insights (33–35). A SNP was mapped to a gene when at least one of the following conditions was met. First, the SNP was located on the gene body or up to 10 kb apart from the gene. Second, the SNP was an eQTL of a gene in at least one available tissue type of GTEx v8. Third, the SNP was located at least one of the known chromatin interaction regions in FUMA. A detailed setup for FUMA annotations is shown in Table 1. The regional association plot was generated by R.

**Table 1:** The barrier genes that overlapped with significantly suggestive variants of GWAS results in PTB.

**Study**	**Gene_name**	**Category_barrier**	**Subgroup (in the PNAS_Maternal study)**	**Associated_variant**	**Variant_chromosome**	**Variant_position**	**Variant_type**	***p*-value**	**Adj-*p*-value**
FINNGEN	*NOTCH1*	Epidermal		rs184109994	9	136598990	intergenic_variant	2.66E-06	
FINNGEN	*NOTCH1*	Epidermal		rs550621781	9	136760614	intergenic_variant	5.05E-06	
FINNGEN	*NOTCH1*	Epidermal		rs572341085	9	137059748	intergenic_variant	2.58E-06	
FINNGEN	*CLDN10*	Intestinal		rs2042214250	13	95431086	upstream_gene_variant	4.07E-06	
PNAS_Maternal	*F11R*	Intestinal	preeclampsia	rs140103079	1	161029167	intergenic_variant	3.88E-05	9.83E-02
PNAS_Maternal	*F11R*	Intestinal	preeclampsia	rs79287702	1	161028641	intergenic_variant	4.08E-05	9.89E-02
PNAS_Maternal	*F11R*	Intestinal	preeclampsia	rs113645076	1	161004237	intron_variant	4.16E-05	9.89E-02
PNAS_Maternal	*MAGI1*	Intestinal	uterine_related	rs112342007	3	65964722	intron_variant	9.02E-08	1.66E-02
PNAS_Maternal	*MAGI2*	Intestinal	uterine_related	rs118052567	7	78965551	intron_variant	4.61E-08	1.04E-02
PNAS_Maternal	*MAGI2*	Intestinal	uterine_related	rs117037980	7	78944071	intron_variant	9.39E-06	6.83E-02
PNAS_Maternal	*MAGI2*	Intestinal	uterine_related	rs112447832	7	79418529	intron_variant	9.47E-06	6.85E-02
PNAS_Maternal	*MAGI2*	Intestinal	uterine_related	rs111472497	7	79362461	intron_variant	1.00E-05	7.00E-02
PNAS_Maternal	*MAGI2*	Intestinal	uterine_related	rs118052567	7	78965551	intron_variant	4.61E-08	1.04E-02
PNAS_Maternal	*MAGI2*	Intestinal	very_early_preterm	rs73369322	7	79087974	intron_variant	4.28E-06	3.28E-02
PNAS_Maternal	*MAGI2*	Intestinal	very_early_preterm	rs10235466	7	79360737	intron_variant	1.36E-05	3.87E-02
PNAS_Maternal	*MAGI2*	Intestinal	very_early_preterm	rs111438787	7	79068804	intron_variant	1.80E-05	6.63E-02
PNAS_Maternal	*MAGI2*	Intestinal	very_early_preterm	rs73369451	7	78826768	intron_variant	1.84E-05	4.36E-02
PNAS_Maternal	*MAGI2*	Intestinal	very_early_preterm	rs9918566	7	79377518	intron_variant	2.25E-05	7.66E-02
PNAS_Maternal	*MAGI2*	Intestinal	very_early_preterm	rs10244234	7	79377972	intron_variant	2.25E-05	7.66E-02
PNAS_Maternal	*MAGI2*	Intestinal	very_early_preterm	rs66961385	7	79380342	intron_variant	2.25E-05	7.66E-02
PNAS_Maternal	*MAGI2*	Intestinal	very_early_preterm	rs58310347	7	79383216	intron_variant	3.88E-05	9.50E-02
PNAS_Maternal	*MAGI2*	Intestinal	very_early_preterm	rs17152233	7	79317828	intron_variant	4.24E-05	9.87E-02
PNAS_Maternal	*MAGI2*	Intestinal	very_early_preterm	rs116162854	7	79315900	intron_variant	8.49E-05	8.66E-02
PNAS_Maternal	*MAGI2*	Intestinal	very_early_preterm	rs7787005	7	79344136	intron_variant	8.49E-05	8.66E-02
PNAS_Maternal	*MAGI2*	Intestinal	very_early_preterm	rs77476937	7	79345313	intron_variant	8.49E-05	8.66E-02
PNAS_Maternal	*MAGI2*	Intestinal	very_early_preterm	rs7811812	7	79346772	intron_variant	8.75E-05	8.78E-02
PNAS_Maternal	*MAGI2*	Intestinal	very_early_preterm	rs78254548	7	79380919	intron_variant	1.02E-04	9.22E-02
PNAS_Maternal	*PARD3*	Intestinal	very_early_preterm	rs74131628	10	34152922	intron_variant	2.03E-07	7.10E-03
PNAS_Maternal	*PARD3*	Intestinal	very_early_preterm	rs12415515	10	34602685	intron_variant	3.19E-05	9.00E-02
PNAS_Maternal	*PARD3*	Intestinal	very_early_preterm	rs7914275	10	34149821	intron_variant	4.11E-05	9.38E-02
PNAS_Maternal	*PARD3*	Intestinal	very_early_preterm	rs60181617	10	34121086	intron_variant	5.35E-05	7.06E-02
PNAS_Maternal	*PARD3*	Intestinal	very_early_preterm	rs73269421	10	34642009	intron_variant	7.48E-05	8.24E-02
PNAS_Maternal	*LAMA4*	Intestinal	cervix_related	rs12198087	6	112213659	3′UTR_variant	1.22E-07	1.69E-02
PNAS_Maternal	*NOTCH1*	Epidermal	preeclampsia	rs73568519	9	136493735	downstream_gene_variant	1.74E-05	6.72E-02
PNAS_Maternal	*GRHL3*	Epidermal	preeclampsia	rs114663417	1	24341917	intron_variant	2.88E-06	2.93E-02
PNAS_Maternal	*GRHL3*	Epidermal	very_early_preterm	rs11799686	1	24349688	intron_variant	1.63E-08	2.68E-03
PNAS_Maternal	*CLDN14*	Intestinal	preeclampsia	rs116414717	21	36458083	downstream_gene_variant	1.83E-05	6.84E-02
PNAS_Maternal	*CGNL1*	Intestinal	uterine_related	rs74797124	15	57491621	intron_variant	7.72E-06	9.05E-02
PNAS_Maternal	*TJP1*	Intestinal	very_early_preterm	rs45608037	15	29707168	intron_variant	2.03E-05	7.01E-02
EGG_ptb	*LRP5*	BBB		rs312778	11	68340864	intron_variant	4.77E-07	
EGG_ptb	*LRP5*	BBB		rs312777	11	68339796	intron_variant	4.04E-09	
EGG_ptb	*LRP5*	BBB		rs4930590	11	68654399	intergenic_variant	4.95E-07	
EGG_duration	*CLDN15*	Intestinal		rs365397	7	102069176	intergenic_variant	4.39E-06	
EGG_duration	*CLDN15*	Intestinal		rs202162	7	101991968	intergenic_variant	9.91E-06	
EGG_duration	*RHOA*	Intestinal		rs11710434	3	49308697	intergenic_variant	2.26E-06	
EGG_duration	*RHOA*	Intestinal		rs6446284	3	49579564	intergenic_variant	9.84E-06	
EGG_duration	*RHOA*	Intestinal		rs62260755	3	49860885	intergenic_variant	7.61E-06	
EGG_duration	*LAMB2*	Intestinal		rs11710434	3	49308697	intergenic_variant	2.26E-06	
EGG_duration	*LAMB2*	Intestinal		rs62260755	3	49860885	intergenic_variant	7.61E-06	

A variant was mapped to a gene by FUMA, and its variant type was annotated by Ensembl Variant Effect Predictor (VEP). The *p*-values and FDR adjusted-*p*-values were obtained from the respective studies.

### 2.5 Ethical statement

The Institutional Review Board of the E-DA Hospital approved the study (EMRP-113-099).

## 3 Results

### 3.1 *NOTCH1* and other barrier genes exhibit suggestive associations with PTB in maternal GWAS datasets

**Figure 2:**
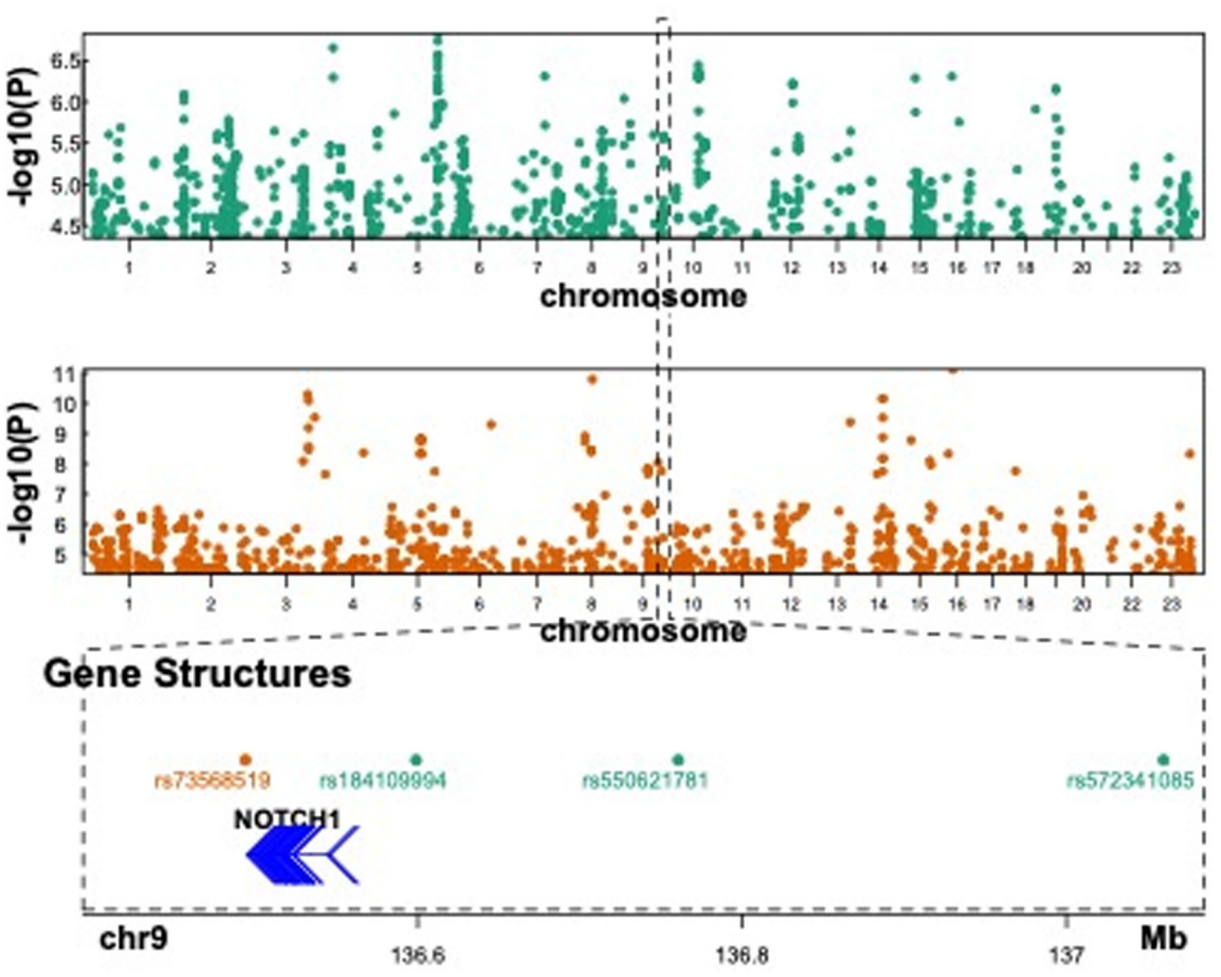
Published GWAS results in PTB and suggestive association at *NOTCH1*. The **upper panel** showed the Manhattan plot of Finngen GWAS in PTB when the *p*-value < 1 × 10^−5^. The **middle panel** showed the Manhattan plot of the maternal GWAS results in PTB with preeclampsia when FDR < 0.1. The **lower panel** showed a focal region in chromosome 9 where significant variants were found in both GWAS results.

There are several types of barrier genes, such as the epidermal barrier, intestinal barrier, blood–brain barrier, and galectin genes. We thoroughly investigated all 201 barrier genes to explore their role in susceptibility to PTB (Supplementary Table S1). A comprehensive screening of various PTB datasets, such as GWAS and WGS, was conducted to identify associations with barrier genes. First, the FinnGen study was used to conduct maternal GWAS analysis on 7,678 PTB and 148,153 term cases. Genetic variants at 4 loci were associated with PTB at suggestive significance (Figure 2, upper panel, and Table 1). Two barrier genes (*NOTCH1* and *CLDN10*) showed potential links to PTB in the FinnGen dataset among the maternal genome. Second, a maternal GWAS analysis was performed on 270 PTB and 521 term cases using the published literature's WGS summary statistics of maternal genomes (21). Genetic variants at 35 loci were associated with PTB at suggestive significance (Figure 2, middle panel, and Table 1). Ten barrier genes (*NOTCH1, LAMA4, F11R, MAGI1, MAGI2, TJP1, PARD3, CLDN14, GRHL3*, and *CGNL1*) showed potential links to PTB in the published literature's dataset among the maternal genome. Notably, the *F11R, NOTCH1, GRHL3*, and *CLDN14* genes were associated with preeclampsia-associated PTB. Third, the EGG meta-analysis was used to conduct maternal GWAS analysis on 15,419 PTB and 217,871 term cases. Genetic variants at 10 loci were associated with PTB or gestational duration at birth at suggestive significance (Supplementary Figure S1 and Table 1). Four barrier genes (*CLDN10, LAMB2, RHOA*, and *LRP5*) showed potential links to PTB in the EGG meta-analysis among the maternal genome (Table 1). Maternal *NOTCH1* was associated with PTB in the FinnGen dataset, which overlapped with findings in the published literature's WGS data. Unfortunately, we found no significant association for *NOTCH1* in the EGG meta-analysis.

### 3.2 Functional annotation of the NOTCH1 SNPs

**Figure 3:**
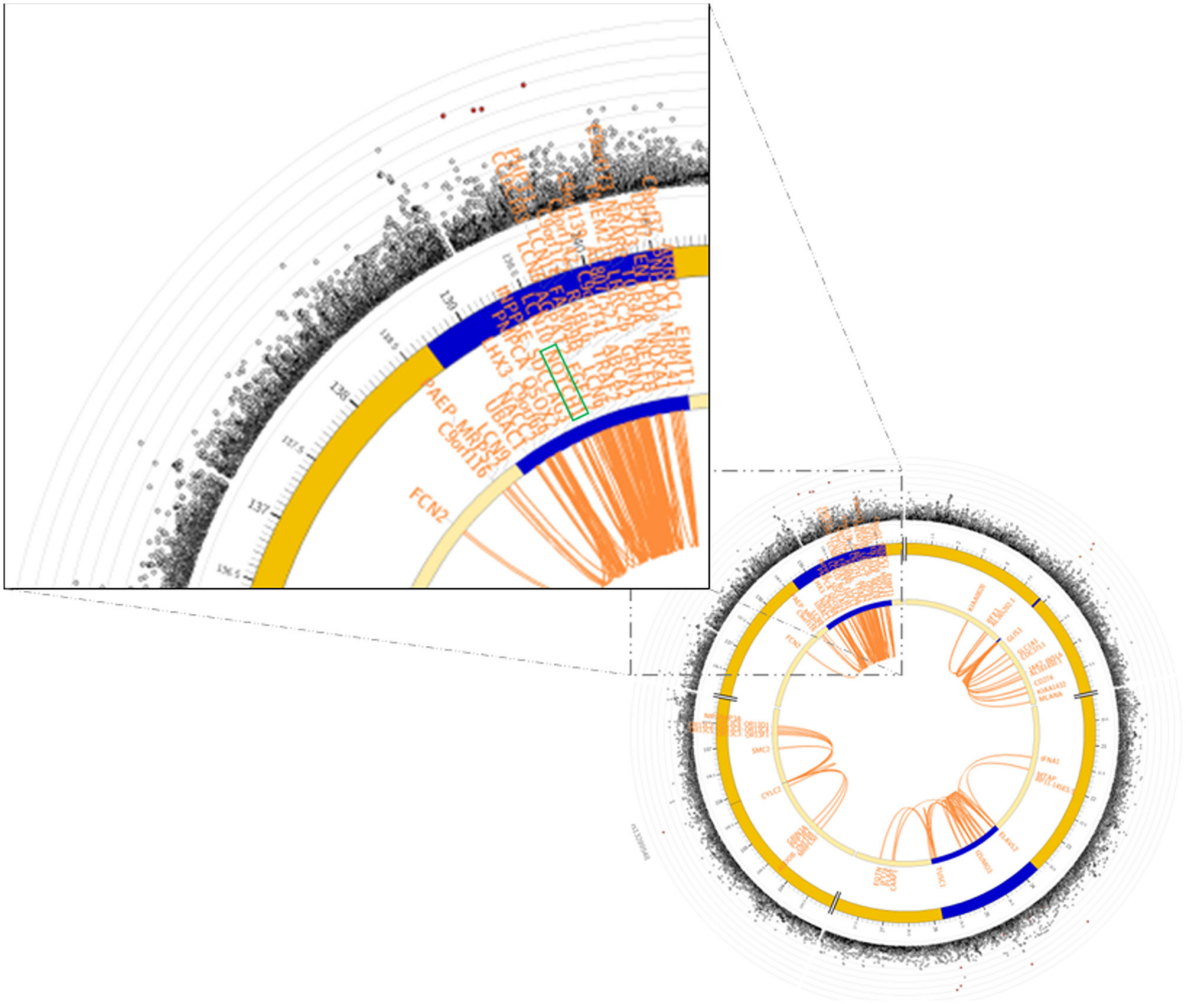
GWAS significant SNPs at *NOTCH1* in Finngen overlapped a known chromatin interaction region. The circos plot on the lower-right side showed the chromatin interaction mapping results of chromosome 9, and the zoom-in on the NOTCH1 region was on the upper-left side. In the circos plot, the Manhattan plot on the most outer layer showed the significance of the SNPs with *p*-value < 0.05, and the SNPs colored in red were in strong LD (i.e., *r*^2^ > 0.8) to one of the independent significant SNPs in the locus. The second and third layers showed the chromosomal locations, and the genomic risk loci were highlighted in blue. The gene(s) being mapped by chromatin interaction were shown within the two layers, and were colored in orange. The links colored orange in the innermost region were chromatin interactions. Further details of the plot were shown in FUMA (https://fuma.ctglab.nl).

Functional annotation of the significant SNPs from 15 imputed genes was displayed in Table 1. Four maternal SNPs (rs184109994, rs550621781, rs572341085, and rs73568519) at the *NOTCH1* locus were associated with PTB. We highlight a genomic region spanning 136–137 Mb (rs184109994, rs550621781, rs572341085, and rs73568519) on chromosome 9 in Figure 2, lower panel. The alleles linked to PTB have not been previously reported. We mapped the associated variants through 3-D chromatin interaction. The circos plot clearly illustrates numerous chromatin interactions between the genomic risk locus and NOTCH1 (Figure 3). Maternal SNPs (rs184109994) lie within the enhancer region of NOTCH1. The mapped placenta is functionally involved in providing nutrients to the fetus, and therefore has implications for its association with the etiology of PTB (Supplementary Table S3). However, according to the GTEx database, whether the four SNPs on NOTCH1 can impact the messenger RNA expression is uncertain.

## 4 Discussion

GWAS has provided valuable insights into genetic risk factors and associated genomic regions for PTB. This is the first GWAS report on identifying maternal barrier genes associated with PTB. Analysis of the maternal genome's GWAS and EGG meta-analysis revealed several barrier genes (*NOTCH1, LAMA4, F11R, MAGI1, MAGI2, TJP1, PARD3, CLDN10, CLDN14, CLDN15, GRHL3, CGNL1, LAMB2, RHOA*, and *LRP5*) associations with PTB. At least two genomic datasets revealed associations of *NOTCH1*.

*NOTCH1* was found to be expressed widely but with different tissue distributions (36). High expression was detected in the intermediate suprabasal layers, whereas low to intermediate expression was detected in lymphocytes in peripheral lymphoid tissues (36). The NOTCH1 signaling pathway in preterm has potential molecular functions, including vascular barrier function, angiogenesis, blood–brain barrier, decidualization, intestinal epithelial barrier, and inflammation (Table 2). First, NOTCH1 signaling is crucial in maintaining vascular stability (Supplementary Figure S2). Upon binding to Delta-like ligand 4, NOTCH1 releases the Notch intracellular domain (NICD), which translocates to the nucleus to induce anti-inflammation and pro-angiogenesis while suppressing endothelial cell proliferation. The non-canonical pathway involves the activation of NOTCH1 to release the transmembrane domain, which forms a complex with VE-cadherin to promote endothelial junction formation (37). Second, NOTCH1 signaling is crucial for decidualization progression (38). The decidua acts as a barrier during pregnancy by regulating trophoblast invasion and the immune response. A previous study indicated that PTB-associated genes RPBJ interact with NOTCH1 according to the STRING tool (21). The NICD regulates the expression of target genes with the DNA-binding protein RBPJ (39). NOTCH1 signaling via RBPJ regulates the expression of ovarian steroid receptor PGR and glucose transporter SLC2A1 during decidualization (40). Decidualization defects result in recurrent pregnancy loss, preeclampsia, preterm labor, and intrauterine growth restriction (40). Third, a previous study indicated that NOTCH1 regulates intestinal epithelial barrier function via balanced tight junction protein complexes and plays a vital role in the mucosal immune response (41). NOTCH1 is essential in early pregnancy, particularly during implantation and placentation. It enables interactions between the endometrium and trophectoderm, regulates extravillous trophoblast invasion, and aids spiral artery remodeling (38, 42). Additionally, it plays a role in placental angiogenesis by guiding vascular branching and maturation (38, 42). Disruption of NOTCH1 signaling has been linked to complications such as preeclampsia, intrauterine growth restriction, polycystic ovary syndrome, endometriosis, adenomyosis, infertility, and endometrial cancer (38, 42). Together, NOTCH1 is crucial for regulating vascular barrier function, angiogenesis, decidualization, intestinal epithelial barrier function, and inflammation during pregnancy, and is critical in preterm delivery (43–45).

**Table 2:** The potential molecular function of Notch1 signaling pathways in preterm.

**Mechanisms**	**References**
Role of Notch1 signaling pathways in vascular barrier function	(37, 108, 109)
Role of Notch1 signaling pathways in blood-brain barrier	(110, 111)
Role of Notch1 signaling pathways in angiogenesis	(112, 113)
Role of Notch1 signaling pathways in inflammation	(114–118)
Role of Notch1 signaling pathways in decidualization	(38)
Role of Notch1 signaling pathway intestinal epithelial barrier	(41)

The tight junction genes, which include F11R, MAGI1, MAGI2, and TJP1, encode proteins that interact with each other according to the STRING tool (46). Tight junction barrier disruption can increase paracellular permeability, allowing luminal pro-inflammatory molecules to activate the mucosal immune system, causing inflammation and tissue damage (47). *F11R*, which encodes the F11 receptor, is a tight junction protein that connects neighboring epithelial or endothelial cells (48). F11R is associated with microscopic colitis (49). In addition, F11R, E-cadherin, occludin, claudin-1, and ZO-1 are abundant in the human endocervix (50). A lack of tight junctions in the lower female reproductive tract allows pathogens and immune cells to move between epithelial cells (51). Furthermore, previous findings indicated that *F11R* is one of the candidate genes for preeclampsia (48, 52). *TJP1* encodes tight junction protein 1, also known as Zonula occludens-1 (ZO-1). TJP1 is a tight junction protein that connects neighboring epithelial cells and provides cellular integrity (53). TJP1 downregulation in IBD impairs mucosal repair and promotes progression (54). In human placental development, TJP1 also plays a crucial role in trophoblast cell-cell fusion and differentiation (55). In addition, a previous study has shown that the downregulation of TJP1 due to inflammation may be a critical factor in the development of PROM (56). *MAGI1* (MAGUK with inverted domain structure-1) is a tight junction protein that connects neighboring epithelial cells or vascular endothelial cells (57, 58). MAGI-1 and its interacting proteins localize to the tight junctions of epithelial cells, resulting in enhanced epithelial integrity (57). *MAGI-1* is associated with Crohn's disease (CD) and microscopic colitis (49, 59). In addition, MAGI1 is crucial for adherens junction maturation and cell-cell adhesion mediated by VE-cadherin (58). It regulates vascular functions like permeability, NO production, and angiogenesis (57). *MAGI2* (MAGUK with inverted domain structure-2) plays a crucial role in maintaining the barrier function of the kidney (60). In addition, previous studies indicated that significant associations were found between *MAGI2* and celiac disease, IBD, CD, as well as ulcerative colitis (UC) (46, 61–63).

The FinnGen, EGG, and WGS data from published literature indicate that the maternal CLDN gene family (*CLDN10, CLDN14*, and *CLDN15*) was associated with PTB. Claudins in paracellular channels have three types of selectivity: anion, cation, and water (64). *CLDN10, CLDN14*, and *CLDN15* are members of the claudin family associated with tight junctions (65). CLDN proteins are associated with regulating the differentiation of the intestinal epithelium (66). *CLDN10* is associated with HELIX syndrome (67). CLDN14 acts as a barrier to cations in epithelial cells and is associated with non-syndromic hearing loss and hypercalciuric nephrolithiasis (68–71). The formation of CLDN15-based tight junctions plays a pivotal role in regulating the microenvironment of the small intestine, particularly in controlling ion conductance and ensuring normal-sized morphogenesis (72).

Our data indicate that the maternal laminin family (*LAMA4* and *LAMB2*) was associated with PTB. *LAMA4*, which encodes the laminin subunit alpha 4, is vital in promoting cell migration, proliferation, apoptosis, angiogenesis in trophoblast cells, microvessel maturation, and maintaining endothelial cell tightness (73–75). LAMA4 is one of the isoforms of laminin that regulates the maturation and function of the blood-brain barrier (76). Previous studies have shown that *LAMA4* is implicated in regulating the onset and progression of preeclampsia (73, 77, 78). *LAMA4* is a critical factor in the differentiation and invasion of trophoblasts (78). Laminin β2 (*LAMB2*) is a crucial component present in the intestine, glomerular basement membrane, neuromuscular junctions, and various ocular structures, and it is associated with Pierson syndrome (79).

Additionally, some genes related to cell junctions (*PARD3* and *CGNL1*) have been identified, and some genes associated with barrier functions (*RHOA, GRHL3*, and *LRP5*) have also been linked to PTB. *PARD3* (Par-3 Family Cell Polarity Regulator, also known as PAR-3) is a regulator of cell polarity in tight junctions of epithelial cells. Previous studies suggest that *PARD3* is linked to IBD, celiac disease, CD, and UC (63). *CGNL1* (cingulin-like 1) co-localizes with actin filament bundles, suggesting it could be a key modulator linking intercellular junction assembly to actin cytoskeleton-regulated morphogenesis in angiogenesis (80). *RHOA* is essential for endothelial barrier function (81). RhoA regulates signal transduction, actomyosin dynamics, cell shape, adhesion, division, migration, trafficking, and proliferation (81). A previous study revealed a significant increase in GTP-bound RHOA in the myometrium of women undergoing spontaneous preterm labor (82). Grainyhead-like 3 (*GRHL3*) is essential for maintaining skin barrier function and epidermal proliferation (83). *GRHL3* is linked to Van der Woude Syndrome and Neural tube defects (84–87). Low-density lipoprotein-related receptors 5 (*LRP5*) is a co-receptor of Wnt/β-catenin signaling and plays a significant role in retinal vasculature development (88). A previous study indicated that intronic variants of the *LRP5* gene may be associated with obesity due to their impact on the WNT signaling pathway or lipid metabolism (89).

### 4.1 Clinical and research implications

The microbiome is a multifaceted characteristic influenced by various factors, such as host genetics and the environment (90). Given the mechanistic link between barrier defects, dysbiosis, and inflammation, it is tempting to speculate that barrier dysfunction leads to microbiota dysbiosis, with resultant inflammation and PTB. For instance, dysregulation of the barrier results in microbiota dysbiosis. The current gut–placenta axis hypothesis indicates that microbiota-derived metabolites or pathogenic microorganisms may pass from mother to fetus through the placenta and harm the fetus (91). Additionally, intrauterine infection leading to PTB is a result of pathogens ascending from the vagina (92). Finally, vaginal dysbiosis is linked to bacterial vaginosis, PTB, premature membrane rupture, and chorioamnionitis (93, 94). Although our current study does not include microbiome analysis, given these facts, it is worthwhile to investigate the relationship between tight junction genes (*F11R, MAGI1, MAGI2*, and *TJP1*), dysbiosis, and PTB in future studies.

Ambient air pollution, including PM_2.5_, nitrogen dioxide (NO_2_), and O_3_, is associated with adverse perinatal outcomes, including PTB (95–97), which may be attributed to inflammation (98), placental inflammation, and reduced blood flow (99, 100). Additionally, air pollutants can disrupt the epithelial barrier, contributing to respiratory diseases such as asthma and chronic obstructive pulmonary disease (101). These findings parallel our observations of barrier-gene dysregulation in PTB, suggesting that genetic susceptibility in barrier-related pathways may exacerbate the inflammatory effects of environmental exposures. Such dysfunction of the barriers could make the maternal–fetal interface more susceptible to inflammation caused by pollutants, thereby increasing the risk of PTB. This underscores a potential interaction between genetic factors and environmental influences in PTB.

Preeclampsia and PTB may be linked to maternal barrier defects, indicating that they may share similar mechanisms (21). For example, endothelial dysfunction is prevalent in preeclampsia, characterized by barrier disruption and reduced vasodilatory capacity, which can lead to PTB (102). VE-cadherin is a key protein in endothelial cells that regulates vascular permeability and cell–cell contacts. If it is disrupted, the endothelial barrier function may be compromised, causing inflammation and other cellular dysfunctions (103, 104). Our findings indicate a correlation between these genes and PE, as well as VE-cadherin, such as *NOTCH1, LAMA4, MAGI1*, and *F11R*. Unfortunately, we could not find variants at the *NOTCH1, LAMA4, MAGI1*, and *F11R* loci associated with PE in the FinnGen database (Supplementary Table S2).

### 4.2 Strengths and limitations

The major strength of the study is its examination of the potential association between maternal barrier genes and PTB. We identified several maternal barrier genes in different datasets. Our study has provided new insights into the dysfunction of the barrier, which can disrupt microbial homeostasis, leading to inflammation and PTB.

We have identified certain limitations in our study. First, our data were unable to differentiate between medically indicated PTB and spontaneous PTB (preterm pre-labor rupture of membranes and idiopathic PTB). Second, we did not observe a significant association for *NOTCH1* in the EGG meta-analysis. This could be explained by the large sample sizes, which have resulted in modest discoveries for PTB due to small effect sizes (2, 105). In addition, concerns have been raised about the suitability of meta-analysis methodologies in GWAS due to preterm heterogeneity observed among studies investigating the same trait (2, 105). The factors contributing to variability among studies can differ significantly due to variations in measurement techniques and research methodologies, the incorporation of diverse ethnic populations, exposure to varied environmental influences, and the use of different genotyping platforms (2, 105, 106). Third, the three significant *NOTCH1* variants are located in UTR regions and have no direct effects attributed to these SNPs.

## 5 Conclusion

Early detection of the risk of PTB can reduce the global burden of adverse neonatal outcomes (107). This study confirms that genomic constitutions may contribute to the risk of PTB in women before or during pregnancy. Our findings, based on GWAS, provide novel insights into maternal barrier function and PTB. Further investigations are warranted to replicate the association between barrier genes and PTB and to explore the mechanisms of barrier defects in the pathogenesis of PTB.

## Data Availability

The original contributions presented in the study are included in the article/Supplementary material, further inquiries can be directed to the corresponding author.
