# Supplementary material for: Barrier genes are associated with preterm birth
Source: Front Med (Lausanne). 2025 Jun 23;12:1580877. doi: 10.3389/fmed.2025.1580877 (PMC12230043; doi:10.3389/fmed.2025.1580877)
Supplement: Supplementary file 4 [file Data_Sheet_1.docx]

Supplementary Material


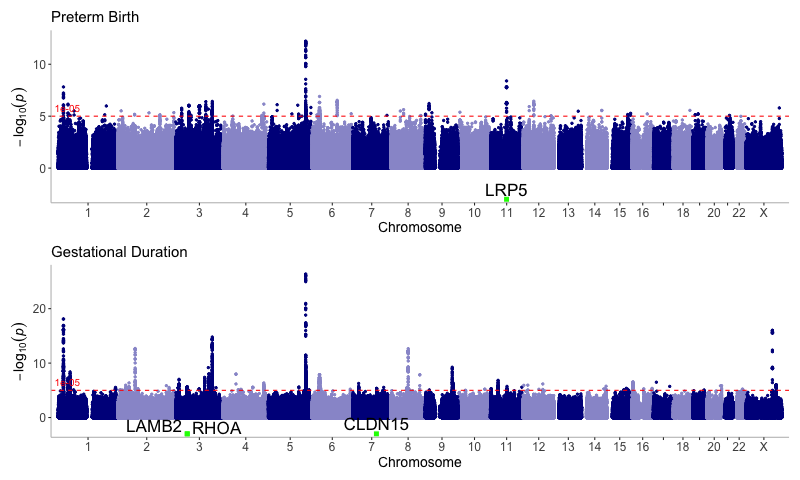


**Supplementary Figure 1.** Published maternal GWAS results in the EGG study and the barrier genes (highlighted in light-green) that overlapped the suggestive significant variants. The threshold of suggestive significance was p-value<1x10^-5^ . The plot was generated by R.


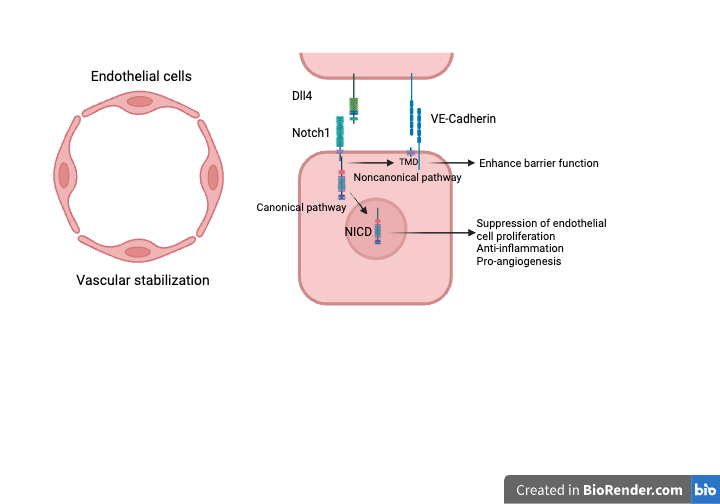


**Supplementary Figure 2.** **A model for the Notch1 signaling in the endothelial barrier.** Notch1 signaling is essential for vascular stabilization. Canonical pathway: upon Notch1 binding to Delta-like ligand 4 (Dll4), the Notch intracellular domain (NICD) is released and translocated to the nucleus to induce anti-inflammation and pro-angiogenesis but suppress endothelial cell proliferation. Non-canonical pathway: Notch1 activation is essential for releasing transmembrane domain (TMD), which plays a vital role in forming a complex with VE-cadherin to enhance endothelial junctions. The figure was created using BioRender.
